# Digital Marketing of Unhealthy Foods and Non-alcoholic Beverages to Children and Adolescents: A Narrative Review

**DOI:** 10.1016/j.cdnut.2025.104545

**Published:** 2025-01-13

**Authors:** Gabriela Fretes, Paula Veliz, Ana Maria Narvaez, D’Arcy Williams, Romain Sibille, Maaike Arts, Jef L Leroy

**Affiliations:** 1 Nutrition, Diets and Health Unit, International Food Policy Research Institute, Washington, DC, United States; 2 United Nations Children’s Fund, Latin America and the Caribbean Regional Office, City of Knowledge, Panama, Republic of Panama; 3 United Nations Children’s Fund, New York, NY, United States

**Keywords:** digital marketing, unhealthy foods, children, adolescents, food policy

## Abstract

With growing access to electronic devices and time spent online, the food and beverage industry increasingly uses digital media to market unhealthy foods and non-alcoholic beverages (high in unhealthy fats, sugars, and/or salt and often highly processed) to children and adolescents. We conducted a narrative review of the global evidence on digital marketing of these foods and drinks and studied policies and regulations in Latin American and Caribbean (LAC) countries. Evidence was limited to a few high, upper-middle and lower-middle income countries where children and adolescents were found to be extensively exposed to the digital marketing of unhealthy foods and non-alcoholic beverages and this exposure increased with age. A wide range of purposefully designed marketing techniques were used. Exposure to the digital marketing of unhealthy foods appears to be followed by increased consumption, but the quality of the evidence was limited. Accurate assessment of exposure was a shortcoming in most studies. Stronger evidence will require studies with more rigorous designs that minimize confounding and objectively quantify individual exposure. Mandatory comprehensive policies are needed that limit exposure of children and adolescents to the marketing of unhealthy foods and non-alcoholic beverages irrespective of the medium or platform they use. The experience of LAC countries may provide insights for the development of effective policies in other countries. Novel technologies that can be used by governments to monitor digital marketing regulations are needed.

## Introduction

The last decades have seen a steady increase in the prevalence of childhood overweight and obesity across most regions in the world [1]. This situation is concerning, considering that overweight and obesity are risk factors for the development of noncommunicable diseases (NCDs) such as diabetes, cardiovascular diseases, and certain types of cancer [2]. Unhealthy weights and NCDs are inextricably related to poor diets. Children’s and adolescents’ diets are in part shaped by the food environment—the physical, economic, political, and sociocultural contexts through which consumers interact with food systems to purchase, prepare, and consume food [3]. Food environments increasingly offer cheap unhealthy foods and non-alcoholic beverages (that is, high in unhealthy fats, sugars, and/or salt and often highly processed) that are aggressively marketed [[4], [5], [6]].

Marketing, a key element of the food environment, is present in environments frequented by children and adolescents such as schools and sports clubs, on television, and in the digital environment that includes websites, social media platforms, and online games [7,8]. Because children and adolescents have greater access to electronic devices and spend more time online than a decade ago [9,10], the food and beverage industry increasingly uses digital media as a complement to traditional media such as television to maximize marketing reach. Marketing on digital media has been deemed cheaper relative to marketing on traditional media [11,12]. Online marketing targeting children uses a wide range of strategies to maximize impact (Box 1) [[13], [14], [15], [16]]. These include graphics and visuals designed to appeal to children; cartoons and brand equity characters; influencers; humor, fun and fantasy; movie and sports celebrity endorsements; engagement through “likes, comments, and shares;” and tie-ins with competitions and entertainment events [13]. Digital ad spending overall accounted for more than half of total media ad spending in 2021 globally [17,18]. Global food ad spending on social media such as TikTok, Facebook, and Instagram has significantly increased since 2020 and is projected to continue rising [19].

Box 1 **Digital marketing terminology**

Branded hashtag challenges: Common on TikTok and created and paid for by brands and usually analyzed by influencers. They encourage sharing a specific action or challenge for others to copy.

Branding: A marketing feature that provides a name or symbol that legally identifies a company, a single product, or a product line to differentiate it from other companies and products [13].

Engagement: Online interaction or conversation through likes, comments, shares, clicking on links, branded hashtag challenges that encourage the creation of user-generated content, etc. [14].

Hashtag: Words or phrase following the hash (#) symbol. Used to categorize and track content in microblogging (e.g., Twitter) and photo-sharing (e.g., Facebook, Instagram, TikTok). For example, #comfortfood.

Influencer: An internet personality whose opinions and recommendations influence their (large) subscriber base usually through a personal channel (e.g., on YouTube) [15].

Licensed character: Character owned by one company and licensed to another company for promoting a product [13].

Marketing: Commercial communication designed to increase the recognition, appeal, and consumption of particular products or services [13,16].

Brand-owned account: Accounts or websites that are owned by brands, e.g., brand website, social media postings, and email marketing [14].

Persuasive marketing techniques: The use of competitions and giveaways, celebrity endorsement, cartoon characters, product demonstration, peer popularity appeal, etc.

Product placement: The use of a message, brand logo, or product in a visual or graphic medium such as television programs, films, music, videos, video games, advergames [13].

User-generated content: Content such as photos or videos created by users that are made publicly available online and are not explicitly part of a marketing campaign. Creators may not be remunerated but instead seek to express themselves, connect with peers, or achieve fame [14].

Alt-text: Box 1

Changes in ad spending combined with increased hours of engaging with digital media have dramatically increased exposure to marketing of unhealthy foods and non-alcoholic beverages among children and adolescents [20,21]. This problem is compounded by the rise in online engagement of children and adolescents because of the COVID-19 pandemic [22]. Exposure of children and adolescents to such marketing is at odds with their right to adequate nutrition, health, privacy, and information [23,24].

A 2022 systematic review and meta-analysis documented that child and adolescent exposure to food and non-alcoholic beverage marketing was associated with *1*) increased dietary intake; *2*) greater odds of choosing a marketed food item; and *3*) increased preference for the marketed food [6]. The included evidence was limited, however, for low- and middle-income countries (LMICs), for adolescents, and for digital media. Another systematic review found that social media exposure was associated with increased consumption frequency of fast food, unhealthy snacks, and sugar-sweetened beverages [25]. The review was limited to social media, leaving out other forms of digital marketing such as websites and online games that are frequently used by children and adolescents. Since the publication of these reviews, several new studies have been conducted on how children’s and adolescents’ decisions (that is, convincing caregivers to buy foods, and buying and consuming foods themselves), food preferences, and diets are influenced by digital marketing in LMICs.

We conducted a narrative review of the evidence on the exposure to and power of digital marketing of unhealthy foods and non-alcoholic beverages to children and adolescents. Exposure refers to the reach and frequency of a particular message and depends on the communication channels, times, and settings in which children encounter marketing [13]. Power is the extent to which the message achieves its communication objectives. It depends on the content of the message and in particular on the creative strategies used. These include marketing techniques such as the use of cartoon characters, celebrity endorsements, promotions, and games. The effect of marketing depends on both exposure and power [13]. The initial focus of the review was on Latin American and Caribbean (LAC) countries. Because most of the evidence had a broader geographic focus, we expanded the scope to include studies from any region of the world. The second objective was to review existing policies and regulatory frameworks on the digital marketing of such foods and beverages with a focus on LAC.

## Methods

### Data sources, search strategies, and inclusion criteria

PubMed database and Google Scholar were searched for peer-reviewed articles, systematic reviews, and grey literature published in English and in Spanish between January 2012 and July 2024. We limited our search to the last 12 y based on previous narrative and systematic reviews that observed that most studies that assessed digital marketing were conducted in the past decade. Earlier studies mostly focused on other media such as TV [6,7,26]. We considered articles that assessed digital marketing exposure in children and adolescents ages 2–18 y, the power of marketing, and the effect of this marketing on outcomes such as food preference, food choice, and food intake. The English and Spanish search terms were based on 2 recent systematic reviews (Supplemental Table 1) [6,26]. Studies were included if they met a prespecified set of criteria (Supplemental Table 2). We conducted a search of the reference list of existing reviews to identify key articles not identified through the database search. The database searches were complemented with information on marketing policies from the World Health Organization (WHO) Global Database on the Implementation of Nutrition Action [27], the Global Obesity Observatory [28], and the World Cancer Research Fund International’s NOURISHING database [29] using the same criteria. Unhealthy foods and non-alcoholic beverages were defined as those that are harmful to health, that is, high in unhealthy fats, sugars, and/or salt and often highly processed. Examples include sugary drinks, salty snacks, sweets, and fast food [30]. The WHO regional offices in Europe and LAC recommend against the marketing of these foods to children [31,32]. Lastly, we reviewed existing food marketing policies from 5 LAC countries (Argentina, Brazil, Chile, Peru, and Mexico) that have recently implemented marketing regulations, including on digital media. We screened policy documents (that is, laws or decrees) posted on official websites for key terms such as digital marketing, internet, social media, or “any” media.

We identified a total of 51 articles, of which 21 were included in this review. A total of 14 articles were excluded because they only evaluated traditional media (*n =* 10) and studied commercial foods for infants and young children or energy drinks (*n =* 4). The remaining articles were excluded because they were part of the included systematic reviews (*n =* 16). We critically appraised each article for methodological quality using the Quality Criteria Checklist [33]. Studies were categorized as positive, neutral, or negative with respect to their overall quality [33]. Positive indicates that the study clearly addressed issues of inclusion/exclusion, bias, generalizability, and data collection and analysis; negative indicates that these issues were not adequately addressed; and neutral indicates that the study was neither exceptionally strong nor exceptionally week. We downgraded the quality rating to negative if a study limitation was so fundamental that it undermined the validity of the study. Quality assessments were conducted by one author (GF) and reviewed by a second author (JLL).

The included articles were tabulated using the following categories: author, year, study topic, design, location, sample, age group, objective, methods, results, potential limitations, and study quality. Policies were tabulated using the following categories: country/year created, name of law or decree, key features, institution in charge of marketing monitoring and evaluation, and potential limitations.

We organized the write-up of the evidence as follows: *1*) exposure to and power of digital marketing of unhealthy foods and non-alcoholic beverages in children and adolescents; *2*) association between exposure to and power of digital marketing and children’s and adolescents’ outcomes; and *3*) policies on digital marketing of unhealthy foods and non-alcoholic beverages.

## Results

Of the 21 studies included in this review, 2 were systematic reviews [6,25], 1 was an experimental study [that is, randomized controlled trial (RCT)] [34], and 18 were observational studies of which 17 were cross-sectional [14,20,21,[35], [36], [37], [38], [39], [40], [41], [42], [43], [44], [45], [46], [47], [48]], and 1 longitudinal [49] (TABLE 1, TABLE 2). Sixteen studies reported on exposure to and/or power of digital marketing of unhealthy foods and non-alcoholic beverages (Table 1) and 5 on associations between exposure to and/or power of digital marketing and children’s and adolescents’ outcomes (Table 2). Most of the studies analyzed were global in scope, with only some conducted in upper-middle and lower-middle-income countries (China, India, Malaysia, Mexico, and Philippines). Different digital platforms were studied (Table 3) [[50], [51], [52], [53], [54], [55]].

**TABLE 1:** Summary of studies on exposure and/or power of digital marketing of unhealthy foods (*n =* 16).

Study (ref), year	Study topic; design; location	Sample, age group	Objective	Methods	Results	Potential limitations^1^	Quality^1^ [33]
Observational studies							
Alruwaily et al. [35], 2020	Exposure, Observational, Cross-sectional, Global	*n =* 418 YouTube videos from channels of 5 most-watched kid influencers aged 3–14 y in the United States as of July 2019. On each channel, 50 videos with the most views and 50 more recent videos with food and beverages in the thumbnail were identified (expected sample 500 videos)	+ Determine the frequency of promoting (un)branded foods and drinks in kid influencer YouTube videos + Assess the nutritional quality of food and drinks shown	+ Content analysis of YouTube videos was conducted manually + Foods and drinks were classified into healthy and unhealthy according to their nutritional quality using the UK Nutrient Profiling Model (NPM)	+ 179 of 418 videos showcased food and/or drinks a total of 291 times + 90.34% of food and/or drinks were unhealthy branded items (e.g., McDonald’s)	+ Sampling unclear: the final sample was 418 instead of the expected 500 videos. Not clear which channels had fewer videos than expected + Not clear how the videos that had a food or beverage in the thumbnail were selected + Because of purposive sample based on thumbnails, videos not representative of videos children watch	Negative
Bragg et al. [49], 2020	Exposure and power, Observational, Longitudinal, United States	*n =* social media accounts of 200 food and beverage brands with highest ad expenditure in the United States between 2007 and 2016, and *n =* 2000 posts of a subset of 20 brands with the highest number of followers present on Facebook, Instagram, Twitter, Tumblr or Vine, between 2007 and 2016	+ Quantify the frequency of food and beverage ads on social media + Examine advertising techniques on 5 social media platforms (Facebook, Instagram, Twitter, Tumblr, or Vine) and the nutritional quality of promoted products + Assess the association between marketing themes and techniques	+ Content analysis of social media accounts (Facebook, Instagram, Twitter, Tumblr, and Vine) of 200 food and beverage brands was conducted manually + Analysis of marketing themes and healthfulness of products in 2000 posts of a subset of 20 brands. Nutritional quality was evaluated using quantitative Nutrient Profile Index (NPI) scores (≥64: healthy) + Multilevel regressions (with a random intercept for brand level variability) of marketing themes (e.g., adolescent socializing) on type of interactive tools (e.g., posts, hashtags) (no adjustments)	+ Between 2007 and 2016, a total of 12.4 million posts were generated across the 5 platforms included in the study + Unique social media features such as geo-tags appeared in 75% of posts. Celebrities were featured in 20.4% of posts and a third of posts used interactive tools such as hashtags. The average NPI score of posts showing food and snack products was 54.1 + Interactive tools were more likely to be found in posts featuring adolescents compared with those featuring adults	+ Results table for the association between marketing themes and techniques not shown + Content viewed by authors (in this particular case posts on Facebook, Instagram, Twitter, or Vine) may be different from what is viewed by young people (i.e., content is based on personalized algorithms). It is not clear if authors used their own accounts or new accounts to access the posts	Negative
Bragg et al. [37], 2017	Exposure and power, Observational, cross-sectional, multi-country (China, Germany, India, Mexico, Philippines, United States)	*n =* 406 screenshots of 18 websites and pages that could be accessed with one click from the home page: Coca-Cola, McDonald’s and Kentucky Fried Chicken in China, Germany, India, Mexico, Philippines, United States collected in June 2016	+ Identify the types of marketing techniques used on fast food and beverage company websites + Examine the nutritional quality of the products marketed + Compare the marketing strategies and nutritional quality of products marketed across high-income (HICs), upper-middle income (UMICs) and lower-middle income countries (LMICs)	+ Qualitative analysis of marketing themes used on 18 websites’ home pages and every page that could be accessed with one click from the home page + Nutritional quality of products was evaluated using quantitative Nutrient Profile Index scores or food categories (e.g., fried, fresh) in the absence of nutritional information for the food or beverage + Chi-squared tests and Fisher’s exact test for differences across countries	+ The marketing techniques used included product images, promotional material directed to children, promotions referring to a charity and promotions referring to sports + Diet products and fresh meals were more frequently promoted in HICs compared with UMICs and LMICs. Fried dish promotion was similar across countries + Marketing directed to children (e.g., cartoon characters, images of children) was more frequent in UMICs compared with HICs and LMICs + Promotions that mention benefits to a charitable organization/cause were more frequent in LMICs compared with HICs and UMICs	+ Representativity of marketed products might be affected by seasonality (i.e., data collected only in June 2016)	Neutral
Brooks et al. [14], 2022	Exposure and power, Observational, Cross-sectional, Global	*n =* 539 TikTok videos posted on the accounts of 16 food and beverage brands (top 5 in the confectionary, snacks, drinks and chained foodservice categories) ≤30 June 2021 (expected 20 brands∗) ∗Two brands were in the top 5 of both the snacks and confectionary categories and two brands did not have a TikTok account. Therefore, the final number of brands for analysis was 16 food and beverage brands (confectionary = 3, snacks = 3, drinks = 5, and chained foodservice = 5)	Examine the use of marketing on food and beverage brand-owned accounts on TikTok	+ Content analysis of all videos posted on the TikTok accounts of 16 food and beverage brands was conducted manually	+ Videos posted by food and beverage brands mostly featured branding (87%), product images (85%), engagement (31%) and influencers (25%) + Engagement (though branded hashtag challenges) encouraged user-generated content featuring brand and products + The number of views of all videos with a hashtag of a branded hashtag challenge ranged from 12.7 million to 107.9 billion	+ Reach and engagement could have been influenced by the presence of bots (automated accounts) and therefore, overestimated + Content viewed by authors (in this particular case a selection of TikTok videos) may be different from what is viewed by young people (i.e., content is based on personalized algorithms). Authors created a new account that have not engaged with any videos previously	Negative
Brownbill et al. [38], 2018	Exposure and power, Observational, Cross-sectional, Australia	*n =* 466 Facebook posts by 6 of the most popular sugar-sweetened beverages (SSBs) Facebook pages, identified through Socialbakers from January to June 2015. The top 2 Australian specific pages with the highest number of followers were selected for each category: soft drinks (Coca-Cola and Pepsi), sports drinks (Gatorade and Powerade) and energy drinks (Red Bull and Monster Energy)	Assess how SSBs are marketed to young people in Australia through content posted by brands on Facebook pages	+ Content analysis of Facebook SSBs posts was conducted manually + Quantitative analysis for explicit marketing techniques, posts, and beverage type + Thematic analyses for implicit marketing messages	+ There were around 1.9 million engagements across the 6 SSBs Facebook pages (i.e., likes, comments, shares) + Posts were mostly photos (64%) or videos (34%) and encouraged followers to do something (70%) + Three themes were identified for one soft drink brand (i.e., Coca-Cola): fun, happiness and friendship for young people. For the rest of the brands (i.e., Pepsi and all sports and energy drinks), different themes were identified: sporting prowess, masculinity, and the outdoors	+ Criteria to select posts on Facebook pages unclear + Content viewed by authors (in this particular case posts on Facebook pages) may be different from what is viewed by young people (i.e., content is based on personalized algorithms). It is not clear if authors used their own accounts or new accounts to access the posts + Sample selection was made considering number of followers of each account. However, it is unknown who the followers are and therefore, it is difficult to know if all posts assessed were targeted to young people + Representativity of marketed products might be affected by seasonality (i.e., only measured 6 mo of the year)	Negative
Coates et al. [39], 2019	Exposure and power, Observational, Cross-sectional, United Kingdom	*n =* 380 YouTube videos uploaded by 2 influencers popular with children ages 5–15 y. Study was conducted between January and December 2017	Explore food and beverage cues featured in YouTube influencer videos popular with children	+ Content analysis of YouTube videos was conducted manually + Cues were classified into healthy and less healthy according to their nutritional quality using the UK NPM + Other cues characteristics evaluated were type, context, brand and presentation	+ 92.6% (*n =* 353) of videos featured food or beverage cues + Among 353 videos, *n =* 3571 food and beverage cues were identified of which almost half were categorized as less healthy + Cakes, fast food, and chocolates were the most commonly featured products + Less healthy food and beverages were frequently presented in the context of eating out (52.9%) + 94% of cues were not specifically presented as part of a marketing campaign (i.e., no ad disclosure)	+ Video content of only 2 influencers was analyzed, not necessarily representative of the overall population of influencers that kids watch	Neutral
Demers-Potvin et al. [21], 2022	Exposure, Observational, Cross-sectional, multi-country (Australia, Canada, Chile, Mexico, United Kingdom, United States)	*n =* 9171 boys and girls∗ from different socioeconomic backgrounds, ages 10–17 y. Study was conducted between November and December 2019 ∗Australia *n =* 1127; Canada *n =* 2869; Chile *n =* 1124; Mexico *n =* 1505; United Kingdom *n =* 1140, and United States *n =* 1406	Examine adolescents’ media engagement, habits and self-reported exposure to unhealthy food and beverage marketing among younger and older adolescents	+ Web survey to assess self-reported exposure to screen-based media, use of social media platforms, location and frequency of exposure to unhealthy food marketing + Exposure was assessed in the past 30 d	+ Chile had the highest reported screen time with 10.2 h/weekday + Instagram was the most popular platform, followed by Facebook and Snapchat + Up to 60% of participants reported having seen unhealthy food and beverage advertising on digital media in the past 30 d + Participants who self-reported more screen time were more likely to report daily exposure to sugary drinks and fast food ads	+ Self-reported exposure could suffer from reporting and recall bias + Participants were sampled from a market research panel using nonprobability-based sampling (i.e., data do not provide nationally representative estimates)	Neutral
Fleming-Milici et al. [40], 2020	Exposure and power, Observational, Cross-sectional, United States	*n =* 1564 boys and girls from different socioeconomic backgrounds, ages 13–17 y. Study was conducted between March and May 2017	+ Measure adolescents’ screen time and their engagement with food and beverage brands on social media + Identify differences in engagement by sociodemographic characteristics	+ Online survey to assess self-reported engagement (i.e., ever liked, shared, followed brands) with food and beverage brands on social media + TV and digital media screen time were measured using validated tools	+ Participants reported 2 h 30 min of TV viewing plus 3 h 23 min of other screen time use on a school day + Most adolescents (70%) engaged with ≥1 food or beverage brand on social media + 53% engaged with ≥1 fast food brand, 45%–50% engaged with sugary drink, candy and snack brands + TV viewers were more likely to engage with brands on social media + Hispanic adolescents were more likely to engage with ≥1 fast food, sugary drink, snack, or candy brand compared with White adolescents	+ Self-reported exposure could suffer from reporting and recall bias + Online survey may include participants who have more access to technology or are more active online, limiting diversity in exposure on social media (self-selection bias)	Neutral
Fleming-Milici et al. [47], 2023	Exposure and power, Observational, Repeated cross-sectional, United States	*n =* 400 videos uploaded by 13 popular United States child-influencer channels on YouTube “made-for-kids.” Videos were identified through Social Blade over two 4-mo periods (1 July to 31 October in 2019 and 2020) and selected using systematic sampling with a random start (200 videos/y)	+ Assess the frequency and types of food-related appearances on child-influencer YouTube videos + Quantify changes in the frequency of food appearances from 2019 to 2020 (following ban on food ads in “made-for-kid”s YouTube videos)	+ Content analysis of YouTube videos was conducted manually + Two-proportion z-tests were used to assess differences in the proportion of food-related appearances each year. Bonferroni corrections were applied	+ 65% of videos had ≥1 type of food-related appearance + Branded food-product appearances were found in 38% of videos and nonbranded food-related appearances were found in 51% of videos + There were no significant differences in the number of appearances between 2019 and 2020	+ Video content of only 13 influencers was analyzed, not necessarily representative of the overall population of influencers that kids watch	Neutral
Nieto et al. [20], 2023	Exposure and power, Observational, Cross-sectional, Mexico	*n =* 347 boys and girls from different socioeconomic backgrounds, ages 6–19 y (children 6–11 y, adolescents 12–19 y). Study was conducted between June and December 2020	+ Determine the extent and nature of children’s and adolescents’ exposure to digital food marketing in Mexico + Examine the healthfulness of marketed food and beverages	+ Parents recorded children’s screen during usual recreational internet activity on devices (smartphone, tablet, laptop or computer) for 45-min: two 15-min recordings during the week and one 15-min recording during the weekend + Content analysis of screen recordings was conducted manually using the WHO screen capture protocol + Participants also answered a self-administered questionnaire on media use, demographics, ownership of devices, etc. + Healthfulness of marketed food and beverages was assessed using the Pan-American Health Organization (PAHO) and the Mexican Nutrient Profile Model (NPM)	+ 69.5% of children and adolescents were exposed to ≥1 instance of food marketing during 45 min of screen time + Participants were exposed to a median of 2.7 food marketing exposures per hour during a weekday and weekend day + 9% of the marketing children and adolescents were exposed to was influencer marketing + The most used marketing strategies were product image (63.1%), packaging image (60.8%) and incentives (60.2%) + 93.3% and 91.3% of the promoted products were items not recommended for marketing to children according to PAHO NPM and Mexican NPM, respectively + Among the most common products promoted were ready-made foods, cakes and sweet biscuits, chocolates/energy bars/desserts and savory snacks	+ Convenience sample with overrepresentation of children and adolescents from high-income households + The inclusion criterion of having Wi-Fi at home may have excluded lower income households + No information about ownership of device used for recording, which could introduce bias considering the targeted nature of marketing using algorithms + Data collection was conducted during the COVID-19 pandemic; results might not be representative of usual marketing conditions	Neutral
Potvin-Kent et al. [42], 2019	Exposure, Observational, Cross-sectional, Canada	*n =* 101 boys and girls from different socioeconomic backgrounds, ages 7–16 y (children 7–11 y, adolescents 12–16 y) Year(s) of data collection not reported	Compare the frequency and healthfulness of food ads on social media seen by children with those seen by adolescents, and to estimate their weekly and annual exposure to such ads	+ Participants used their 2 favorite social media platforms∗ for 5 min each. The 10 min total use were recorded using Tobii Pro Glasses + Participants also answered a self-administered questionnaire on media use, demographics, ownership of devices, etc. + Nutritional quality of marketed food and beverages was assessed using the Pan American Health Organization (PAHO) and the UK NPMs ∗Participants could choose from Facebook, Instagram, Snapchat, Twitter, or YouTube	+ The most commonly used platforms during the study were YouTube, Instagram and Snapchat + 72% of children and adolescents were exposed to food ads although using their favorite social media apps. Adolescents viewed more instances of food marketing per 10-min period compared with children (2.6 vs. 1.4, respectively) + Most of the food ads promoted unhealthy products such as fast food (44%), SSBs (9%), candy and chocolate (7%) + 75% of products were less healthy according to the UK NPM, 85% were ultra-processed, and 97% were excessive in ≥1 nutrient according to PAHO NPM + It was estimated that children and adolescents may be exposed to food marketing 30 and 189 times per week, respectively (1560 and 9828 times annually)	+ Media use for a very short time (i.e., 10 min) is not representative of regular social media use + Small convenience sample may have excluded lower income households + Participant recruitment through community centers may have introduced bias (i.e., participants that attend community centers may be different from the general children and adolescent population)	Neutral
Potvin-Kent et al. [46], 2024	Exposure and power, Observational, Cross-sectional, Canada	*n =* 100 boys and girls from different socioeconomic backgrounds, ages 6–17 y (children 6–11 y, adolescents 12–17 y). Study was conducted in 2022	Compare the frequency, healthfulness and power of food marketing viewed by children and adolescents across all digital platforms	+ Participants were asked to use their smartphone or tablet for 30-min although sharing the screen in an online Zoom session. The WHO screen capture protocol was followed for recordings + Content analysis of screen recordings was conducted manually + Participants also answered a self-administered questionnaire on media use, demographics, ownership of devices, etc. + Nutritional quality of marketed food and beverages was assessed using the Health Canada’s proposed NPM from 2018	+ 51% of children and adolescents viewed 226 instances of food marketing∗ in 30 min. Instances were mostly viewed on social media (83%) + Most of the instances of food marketing promoted unhealthy products such as fast food (22%), salty snacks (11%) and soft drinks (9%) + 89% of the products and brands were less healthy according to the Health Canada NPM + The most frequently viewed marketing techniques were appealing graphic effects (20%), songs/music (13%) and calls to action (11%) ∗An instance of food marketing was defined as any occasion where a branded food product or brand was mentioned or shown on the screen capture	+ Small convenience sample and having access to a computer as inclusion criterion may have excluded lower income households + Self-selection of participants due to recruitment happening through social media ads may have introduced bias + Screensharing may have changed digital behavior	Neutral
Tan et al. [43], 2018	Exposure and power, Observational, Cross-sectional, Malaysia	*n =* 250 most popular YouTube videos targeting children selected through SocialBlade.com. First, 25 most popular child-centric YouTube channels were identified and then, for each channel, the top 10 most-viewed videos were selected to be included in the sample. Recording of videos was for 3 wk in October 2017	Assess food and beverage ads on YouTube videos directed to children in Malaysia	+ A social media analytics site was used to identify the most popular videos targeting children + Ads displayed during the YouTube videos were recorded + Analysis were conducted for type of product marketed and ad format + Food and beverage ads were coded by food category and by the use of persuasive techniques	+ A total of 187 ads were identified + 38% of ads promoted food and beverages + The most common products promoted were dietary supplements (28%), fast food (21%) and candy (17%) + The most common persuasive techniques used were taste appeal unique or new product, animations, fun appeal, and use of promotional characters	+ Content viewed by authors (in this particular case, ads shown) may be different from what is viewed by young people (i.e., content is based on personalized algorithms).Videos were watched with browser on “incognito” mode which may also have affected the ads shown	Negative
Theodore et al. [44], 2021	Exposure and power, Observational, Cross-sectional, Mexico	*n =* 129 social media accounts (Facebook, Twitter, YouTube) and websites of 46 food and beverage products (top 10 in the soft drinks, snacks and fast-food categories in Mexico based on the number of followers/views) (expected = 90 food and beverage products∗). Sampling happened through Socialbakers. Study was conducted between February and May 2017 ∗3 categories × 10 products/brands per platform = 90 potential food and beverage products. *n =* 89 products were identified of which *n =* 43 products were excluded from the study (duplicates, products with no Mexican website, products aimed at adults)	Analyze *1*) the general characteristics, *2*) the use of persuasive techniques, and *3*) the nutritional quality of food and beverage products marketed on digital media in Mexico	+ A social media analytics site was used to identify products promoted to children and adolescents on Facebook, Twitter, and YouTube (all accounts had an attractive element to children such as games, characters, bright colors, etc.) + Active social media accounts and websites were identified for each selected product + Six variables were used to characterize advertisements: product/brand information, media information, association (tie-in with logo or link), characters, incentives, downloads, and digital techniques for engagement (e.g., hashtags) + Nutritional quality of products was evaluated using PAHO nutrient profiling model	+ Soft drink social media accounts had the greatest number of followers on Facebook and Twitter + On YouTube, fast food products had the greatest number of views + The main persuasive techniques used included promotional characters, incentives, hashtags, comment function + 85% of products marketed had high free sugar-content and around 69% high saturated fats	+ Content viewed by authors (in this particular case posts on Facebook, Twitter and YouTube) may be different from what is viewed by young people (i.e., content is based on personalized algorithms). It is not clear if authors used their own accounts or new accounts to access the posts + Representativity of marketed products might be affected by seasonality (i.e., only measured 4 mo of the year)	Negative
Valero-Morales et al. [48], 2023	Exposure and power, Observational, Cross-sectional, Mexico	*n =* 926 posts from 20 food and beverage products and brands present on Facebook, Instagram and YouTube in Mexico, identified through Euromonitor and SocialBakers in September and October 2020	+ Assess the extent and nature of food and beverage marketing on Facebook, Instagram, and YouTube posts in Mexico + Examine the healthfulness of marketed food and beverages	+ Sample selection was made considering number of followers of each account + Content analysis of posts on Facebook, Instagram and YouTube was conducted manually using the WHO screen capture protocol + Healthfulness of marketed food and beverages was assessed using the Mexican Nutrient Profile Model (NPM)	+ Facebook had most posts and engagements (i.e. comments, shares, likes/reactions) + The most used marketing techniques were brand logo (91.7%), packaging image (65.7%), product image (65.3%) and hashtags (58%) + Among the food ads appealing to children or adolescents, 92.6% of products were classified as unhealthy according to the Mexican NPM	+ Criteria to select brand or product posts unclear + Content viewed by authors may be different from what is viewed by young people (i.e., content is based on personalized algorithms) + Data collection was conducted during the COVID-19 pandemic; results might not be representative of usual marketing conditions + Representativity of marketed products might be affected by seasonality (i.e., only measured 2 mo of the year)	Negative
Vanderlee et al. [45], 2021	Exposure, Observational, Cross-sectional, multi-country (Australia, Canada, Mexico, United Kingdom, United States)	*n =* 4,827 male and female parents ages 18–64 y from different socioeconomic backgrounds living with their children ages 0–17 y. Study was conducted in November–December 2017	Compare parental self-reported exposure with marketing of unhealthy foods across different settings overall and although co-viewing TV, movies or online content with their children	+ Web-based survey on self-reported parental exposure to all digital marketing of fast food and sugary drinks and specifically although they watch media with their children in the past 30 d	+ Parents living in Mexico and United States reported exposure to marketing of fast food and sugary drinks on more media channels compared with parents living in the other countries (Australia, Canada and the United Kingdom) + Parents living in Mexico reported more frequent exposure to marketing of sugary drinks when viewing online content with their children compared with the other countries (Australia, Canada, United Kingdom and United States)	+ Self-reported exposure through an online survey could suffer from reporting and recall bias. Measurement could be underestimating the actual exposure given that marketing is oriented to influence the consumer subconsciously + Online survey may include participants who have more access to technology or are more active online, limiting diversity in exposure on social media (self-selection bias) + Differences in parenting across countries may affect accuracy of reporting	Negative

1 For original articles, we considered limitations related to the methodological quality of the study. The same applies to the quality column of the table.

**TABLE 2:** Summary of studies on associations between exposure and/or power of digital marketing of unhealthy foods and children’s and adolescents’ outcomes (*n =* 5).

Study (ref), year	Study topic; design; location	Sample, age group	Objective	Methods	Results	Potential limitations^1^	Quality^1^ [33]
Observational studies							
Baldwin et al. [36], 2018	Association between exposure/power and food intake, Observational, Cross-sectional, Australia	*n =* 417 boys and girls from different socioeconomic backgrounds, ages 10–16 y. Study was conducted during October–November 2014	Examine the association between online behaviors and unhealthy food and drink intake in children	Linear regression models of self-reported unhealthy food and drink consumption score on self-reported online behaviors, adjusting for age, sex, and socioeconomic status	Watching food brand video content on YouTube, purchasing food online, and seeing favorite food brands advertised online were significantly associated with a higher frequency of intake of unhealthy foods and drinks	+ Self-reported online behaviors and intake could suffer from reporting and recall bias + A general interest in unhealthy food by adolescents in the sample may be driving both the exposure and the behavior, which makes the direction of the association difficult to establish + The main nutrition outcome was a food score that was not validated	Negative
Pollack et al. [41], 2021	Association between exposure and consumption and purchasing behavior, Observational, Cross-sectional, Global	*n =* 621 Twitch users mostly male (90%) non-Hispanic or Latino (80%) and White (64%), ages 13 y or older. Sampling happened through Reddit (social news platform). Study was conducted in May 2020	Assess users’ self-reported exposure to food marketing, attitudes, consumption, and purchasing behavior on Twitch	+ Participants were asked to report if they had seen ads for 9 food categories on Twitch. In addition, they also responded to questions about food brands, craving foods, purchasing foods, etc. + Chi-squared tests between product viewership, craving and purchasing by product category, type of user (paying vs. nonpaying) or viewership hours. For significant chi-squared test, a multivariate logistic regression model was analyzed adjusted by age, gender, ethnicity, and race	+ Food and beverage ad exposure on Twitch was reported by 72% of participants + 14% of participants reported to crave the food after observing the ad + The most purchased products after exposure and craving included fast food from chain restaurants	+ Sample included ages 13 and older. Therefore, some young adults (<25 y) were included + Results may suffer from reporting and recall bias: those who remember craving or consuming are also more likely to remember seeing the food + Self-selected sample—not clear who the sample is representative of	Negative
Experiments							
Coates et al. [34], 2019	Association between exposure/power and food intake, Experiment—RCT (between-subjects design), United Kingdom	*n =* 176 boys and girls from different socioeconomic backgrounds, ages 9–11 y. Study was conducted between January and July 2017	Assess the impact of social media influencer food marketing of healthy and unhealthy snacks on children’s food intake	+ Children randomly assigned to watch 1 min of mock Instagram profiles of 2 popular influencers (1 female and 1 male) holding a product: unhealthy (treatment arm 1), healthy (treatment arm 2), and nonfood item (control) + Caloric intake was measured right after participants ate ad libitum a selection of foods (candy, chocolate, carrots, grapes) for 10 min	+ Children who viewed influencers holding an unhealthy snack increased overall caloric intake (+448 kcal) as well as caloric intake from unhealthy snacks (+389 kcal) compared with those in the healthy snack condition and those in the control group + Overall caloric intake and caloric intake from healthy snacks was not affected by viewing influencers holding a healthy snack	+ Trial was not registered + It is not known to what extent the effect of 1-min exposure to mock Instagram profiles reflects the effect of being exposed to real profiles + Not clear to what extent study subjects were successfully blinded. Lack of blinding could have biased the results + Higher intake right after watching influencer could be compensated by lower intake later	Neutral
Reviews							
Boyland et al. [6], 2022	Association between exposure/power and food choice, preference, intake and purchasing behavior, Systematic review and meta-analysis, Global	Systematic review: *n =* 96 studies (boys and girls from different socioeconomic backgrounds, ages 0–19 y) from 22 databases (January 2009–March 2020) Meta-analysis: *n =* 80 studies (19,372 participants), boys and girls from different socioeconomic backgrounds, ages 0–19 y	Quantify the association of food and beverage marketing with behavioral and health outcomes in children and adolescents	+ Inclusion criteria: primary studies (experimental and observational) assessing association of food marketing with food preference, choice, purchasing, and intake + Exclusion criteria: qualitative studies and studies assessing infant formula marketing	+ 19 of 96 studies reported on digital food marketing + 14 of 19 studies were RCTs + All studies (*n =* 19) were conducted in high-income countries + Digital food marketing was associated with significant increases in preference, and intake + No clear evidence of association of digital marketing and food choice (nonsignificant)	+ Based on GRADE (Grading of Recommendations, Assessment, Development, and Evaluations), the quality of the overall evidence found was very low or moderate due to high heterogeneity in study designs and effect measures. GRADE for digital marketing evidence only was not reported	Positive
Sina et al. [25], 2022	Association between exposure/power and food intake, Systematic review, Global	*n =* 35 studies (boys and girls from different socioeconomic backgrounds, ages 2–18 y) from 3 databases (2008–December 2021)	+ Determine how exposure to social media (SM) influences children’s and adolescents’ diets + Identify the underlying explanatory mechanisms and technological features SM that may shape children’s eating behaviors	+ Inclusion criteria: primary studies (experimental and observational) assessing the association between SM and food intake and related behaviors + Exclusion criteria: studies conducted with children with disease, lacking a SM component or not measuring diet-related outcomes	+ 23 of 35 studies were RCTs + Almost all studies (*n =* 29) were conducted in high-income countries + SM exposure was associated with increased intake frequency of unhealthy snacks, fast food and sugar-sweetened beverages, and higher odds of skipping breakfast both in children and adolescents + Among the mechanisms that explain the associations are: *1*) peer influence (adolescents) and parental influence (children) on SM; *2*) food and influencer marketing targeting children and adolescents on SM; *3*) access to SM via smartphones, and *4*) food images on SM may elicit brain responses (area related to attention, memory and reward)	+ Among the RCTs, 19 studies were rated low quality mainly because those delivering the interventions and assessing the outcomes were not blinded to the treatment + Information on key determinants such a socioeconomic status was reported only in 2 RCTs (compromising the external validity) and 3 observational studies (residual confounding) + In observational studies, SM exposure and diet-related outcomes were mostly self-reported and could suffer from reporting and recall bias	Positive

1 Potential limitations have a slightly different definition for original articles and review studies. For original articles, we considered limitations related to the methodological quality of the study. For reviews, we considered limitations related to the methodological quality of the review process, not to the quality of the papers included in the review, and thus the strength of the inference. The same applies to the quality column of the table.

**TABLE 3:** Social media platforms studied in the articles included in this review.

Facebook	Facebook is an online social networking site. Users can post photos, status updates, videos, etc., use real-time web chat, play games, and live stream, among others. They can react to content by using the “like,” “share,” and “comment” options. Each user also can search for and connect with friends and family as well as interact with business pages [50].
Instagram	Instagram is an online photo and video-sharing application and social network platform increasingly used by influencers. Users can follow accounts that interest them. They can also react to content by using the “like,” “share,” and “comment” options and browse using hashtags [51].
Snapchat	Snapchat is a multimedia instant messaging app that allows users to send and receive photos and videos called snaps. Content sharing may be private and temporary or public and stored for retrieval [52].
TikTok	TikTok is a social media platform increasingly used by influencers. Users can record short videos, add filters, text, sounds, and music, and upload them to their profiles. They can also react to content and browse using hashtags [53].
Twitch	Twitch is a live streaming platform increasingly used by influencers. This platform’s reach is international, and it is currently the leading provider of video game live-stream material [54].
YouTube	YouTube is a video-sharing website and app where users can post, view, or share video clips online with anyone able to access the site [55].

### Exposure to and power of digital marketing of unhealthy foods and non-alcoholic beverages

#### Exposure to digital marketing of foods and non-alcoholic beverages

Seven studies analyzed children and adolescent exposure to digital marketing (5 neutral quality; 2 negative quality). Studies in European, North American, and South American countries documented a greater exposure of older adolescents (14–17 y) to unhealthy foods and non-alcoholic beverages advertising, compared with children [21,42,46]. A study in Canada found that adolescents were more exposed than younger children to digital marketing of food when using their favorite social media apps for 5 min (83% compared with 55%) [42] or while browsing the internet for 30 min [46]. In a sample of adolescents aged 13–17 y in the United States, Fleming-Milici and Harris [40] found that 7 out of 10 interacted with ≥1 food and non-alcoholic beverage brand on social media and that 3 out of 10 interacted with 5 or more brands. The authors found that Black and Hispanic adolescents were more likely than non-Hispanic White adolescents to engage with brands [40]. A study using a real-time 45-min capture of online video watching in Mexico showed that 69% of children and adolescents aged 6–19 y were exposed to digital marketing of foods and non-alcoholic beverages [20]. The majority of Twitch users aged 13 y or older surveyed on Reddit (a social news site) recalled observing ≥1 food or non-alcoholic beverage advertisement on the platform [41].

Parents in Mexico and the United States reported greater exposure to the marketing of fast food and sugary drinks on television, radio, and digital media although coconsuming media with their children compared with parents in Australia, Canada, and the United Kingdom [45].

#### Food and non-alcoholic beverage being marketed

Ten studies evaluated the healthfulness of the foods marketed on digital media (5 neutral quality; 5 negative quality). Most food and non-alcoholic beverage marketing directed to children was for items that were high in sugars, saturated fats, and sodium such as sugary drinks, fast food, sweets, cookies, and chocolates [35,39,43]. In Mexico, 85% of products marketed on food and beverage company accounts had high free sugar-content and ∼69% high saturated fats according to the Pan American Health Organization (PAHO) nutrient profile model (NPM) thresholds [44]. In 2 other Mexican studies, 93% of products marketed by the most popular food and beverage brands were classified as unhealthy based on the Mexican NPM [20,48]. Similarly, in Canada, nearly 90% of the products and brands marketed were “less healthy” according to the Health Canada NPM [46]. Another study in Canada assessed the nutritional quality of marketed food and beverages using the PAHO and UK NPMs that determines the healthfulness of a given food or drink based on cut-offs for critical nutrients including saturated fats, total sugars, and sodium per 100 g. Authors found that 75% of products were “less healthy” according to the UK NPM, and 97% were excessive in ≥1 critical nutrient according to PAHO [42]. In the United States, around half of the studied adolescents aged 13–17 y interacted on social media with brands of fast food, sugary drinks, candy, and snacks [40]. Interaction was defined as having liked, shared, or followed any of study brands on social media. Another study in the United States found that the nutritional quality of products promoted in 5 different social media platforms (Facebook, Instagram, Twitter, Tumblr, or Vine) was unhealthy [49].

#### Power of digital marketing of food and non-alcoholic beverages: marketing techniques most used

Eleven studies described the marketing techniques used on digital media (5 neutral quality; 6 negative quality). Most common were branding, product placement, the use of celebrities (including influencers and Youtubers), characters (animated, licensed, branded, athletes, other children and adolescents), sports sponsorships, incentives (prizes, contests, gifts, concerts, giveaways), games, animations, appealing graphic effects, different forms of engagement (hashtags, sharing, commenting and tagging others, challenges), emoticons, and use of geolocators [14,20,36,37,40,[42], [43], [44],^46^,^48^,^49^].

Three studies assessed influencer marketing (2 neutral quality; 1 negative quality). In a study that assessed 418 videos by the 5 most-watched kid influencers in the United States on YouTube in 2019, 179 videos (43%) featured food and/or drinks; and 9 out of 10 of the foods or non-alcoholic beverages were categorized as unhealthy based on the UK NMP [56]. By the time of the study (July 2019), the videos showing foods and/or drinks had been viewed more than a billion times in total [35]. Another study in United States assessed 400 videos uploaded by 13 popular child-influencer channels on YouTube “made-for kids” and found that 65% of videos had ≥1 type of food-related appearance [47]. Most YouTube videos of influencers popular with children in the United Kingdom featured foods or non-alcoholic beverages classified as unhealthy based on the UK NMP [39].

### Association between digital marketing of unhealthy foods and non-alcoholic beverages and children’s and adolescents’ outcomes

Five studies assessed the association between digital marketing and different outcomes: 2 observational studies (both negative quality), 1 experimental study (neutral), and 2 reviews (2 positive). An observational study in Australia reported that watching videos of food brands on YouTube and self-reported exposure to favorite food brands advertised online was significantly associated with a higher frequency of self-reported unhealthy food and non-alcoholic beverage consumption in 10–16-y olds, regardless of age, sex, or socioeconomic status [36]. Self-reported interactions or engagements were associated with greater self-reported consumption of unhealthy foods and non-alcoholic beverages compared with exposure to digital marketing without interaction or engagement [36]. A global study conducted through Reddit (a social news platform) found that 14% of Twitch users who recalled observing ≥1 food or non-alcoholic beverage advertisement on the platform reported craving the product after seeing its ad and 8% reported purchasing the product after exposure and craving [41]. An RCT used mock Instagram profiles of 2 popular influencers in the United Kingdom [34]. Exposure to this mock influencer marketing of unhealthy snacks led to increased unhealthy snack intake immediately after exposure. The marketing of healthy snacks did not affect consumption.

One systematic review concluded that exposure to different forms of marketing, including digital marketing of food and non-alcoholic beverages influenced children’s and adolescents’ food preferences and their diet [6]. In another systematic review, social media exposure was associated with increased intake frequency of unhealthy snacks, fast food and sugar-sweetened beverages, and higher odds of skipping breakfast in children and adolescents [25]. Both reviews rated the quality of the evidence as low.

### Policies on digital marketing of unhealthy foods and non-alcoholic beverages

We reviewed food marketing policies from Argentina, Brazil, Chile, Mexico, and Peru (Table 4) [[57], [58], [59], [60], [61], [62]]. Brazil started mandatory marketing regulations in 2014, and other countries followed in recent years. Most policies were laws or decrees with binding force of law. In all countries, the marketing regulation covered any kind of media, including websites and social media platforms. Three countries (Argentina, Chile, and Mexico) used front-of-package (FOP) warning labeling policies to identify the products that cannot be marketed to children; they also banned the use of cartoon characters. One country (Peru) enforced the use of the same octagonal FOP warning labels in ads of unhealthy foods promoted in all media including the internet. Typically missing from the regulations are which digital media and marketing techniques fall under the regulation, and how monitoring will be conducted to enforce compliance in the digital media.

**TABLE 4:** Summary of policies.

Country (ref), year created	Name of law or decree	Key features	Institution in charge of marketing monitoring and evaluation	Potential limitations
Argentina [57], 2022	Law N° 27,642 “Promotion of healthy eating” with its Decree N° 151/2022 and Resolution 6924/2022	+ Prohibits advertising, promotion, and sponsorship of packaged foods with ≥1 octagonal warning label^1^ that is directed to children and adolescents + Prohibition applies to any kind of media, including traditional and digital media + Food packages with ≥1 octagonal warning label cannot have cartoon characters, caricatures, celebrities, or offer gifts or prizes	+ National Administration of Drugs, Food and Medical Devices (ANMAT in Spanish) and National Entity of Communications (ENACOM in Spanish)	+ Not clear which digital media marketing techniques fall under this regulation + The age of “children and adolescents” not specified + Not clear how enforcement is monitored, especially for digital media
Brazil [58], 2014	Resolution N° 163, 13 March, 2014. “About the abusive nature of advertising and marketing communications aimed at children and adolescents”	+ Considers abusive all advertising and marketing communications of products, services, brands and businesses that use child language, special effects and colors, child music, celebrities, cartoon characters, toys, prizes, games. + It considers advertising and marketing communications on printed media, TV commercials, radio spots, internet, packages, promotions, merchandising, products, public spaces, shows/events as well as the activities inside daycares and schools	+ National Council for Children and Adolescent Rights (CONANDA)	+ Resolution is broad and covers a wide range of products, services and activities + Not clear which digital media marketing techniques fall under this regulation + Not clear how enforcement is monitored, especially for digital media
Chile [59,60], 2015	Law N° 20,606 “On nutritional composition of foods and their advertising” and Decree 13 “Modifies supreme decree N° 977, from 1996, Sanitary Regulatory Code”	+ Prohibits marketing directed to children <14 y of products with ≥1 octagonal warning label^1^ on TV, radio, movie theaters, websites, and social media + Prohibits the use of child appealing techniques such as cartoon characters, toys, and gifts	+ Ministry of Health	+ Marketing of regulated products is not allowed on “any” media. However, it is not clear if the techniques to appeal children are regulated in all media + Children older than 14 y are not protected + Not clear how enforcement is monitored, especially for digital media
Mexico [61], 2022	Decree for which various provisions of the Sanitary Regulatory Code of Products and Services and the General Health Regulatory Code on Advertising are reformed, added, and repealed. September, 2022	+ Prohibits marketing of packaged products with ≥1 octagonal warning label^1^ on broadcast TV channels, cable TV, movie theaters, internet, and other digital platforms + Prohibits the use of cartoon characters, sportsperson, celebrities, mascots, or interactive elements such as digital downloads and games directed to children	+ Secretary of Health + Federal Commission for the Protection against Sanitary Risks	+ Not clear which digital media marketing techniques fall under this regulation + The age of “children” not specified + Not clear how enforcement is monitored, especially for the digital media
Peru [62], 2018	Law N° 30,021, “Law to promote healthy eating for children and adolescents”, and Regulatory Decree Nº 017-2017-SA	+ Marketing directed to children <16 y should not: incentivize the consumption of food and beverages high in sugar, sodium, and saturated fats; use claims about improving physical strength; create a sense of urgency or dependency to acquire the food or beverage; use arguments or techniques that exploit children’s ingenuity, etc. + Octagonal warning labels^1^ must be displayed on ads of packaged products that have excess sugar, saturated fats, trans fats and sodium in a way that it is visible to the consumer in all media including radio, TV, movie theaters, internet, billboards, printed media + Octagonal warning labels^1^ displayed on ads will cover until 15% of the size of the ad	+ Commission for the Supervision of Unfair Competition of the National Institute for the Defense of Competition and the Protection of Intellectual Property (Indecopi)	+ Does not prohibit marketing techniques such as the use of cartoon characters on packaging or on digital media + Children older than 16 y are not protected + Not clear how enforcement is monitored, especially for the digital media

1 Octagonal warning labels are graphic black and white labels placed on the front of the package of foods and beverages that indicate high levels of certain nutrients such as total sugars, sodium, or saturated fats. Shaped like octagons, these labels are designed to be easily recognizable and inform consumers about nutrients to limit in their diets.

There was heterogeneity in the age ranges of the target population. In Brazil, any advertising directed at children under 12 y of age is illegal [58]. Chile prohibits all forms of advertising (including digital ads) of unhealthy foods and beverages and the use of characters, celebrities, athletes, toys, and references to school settings in children and adolescents under 14 y of age [59,60]. In Peru, advertising of unhealthy foods and beverages carries an octagonal warning label but there is no ban on marketing techniques such as the use of licensed cartoon characters or figures on food and beverage packaging [62]. In Argentina, the Law on the Promotion of Healthy Eating includes restrictions on all forms of advertising (that is, traditional, and digital mass media) of foods and beverages that carry ≥1 octagonal warning label [57]. In Mexico, digital marketing regulations were recently added to the existing marketing regulations in school settings, television, and movie theaters [61].

## Discussion

We conducted a narrative review on the exposure to and power of digital marketing of unhealthy foods and beverages on children and adolescents. Evidence was mostly limited to a few high, upper-middle, and LMIC. The majority of children and adolescents in these countries were exposed to marketing of foods and beverages on digital media and this exposure increased with age. Most foods and beverages marketed were unhealthy and included fast food, sugary drinks, candy, and unhealthy snacks. Food and beverage companies used a wide range of marketing techniques purposefully designed to be appealing to children and adolescents. These included the use of celebrities and influencers, animated characters, incentives such as contests and giveaways, and different forms of engagement including hashtags, sharing, commenting and tagging others, and challenges.

Exposure to digital marketing appears to affect subsequent consumption behaviors, but the quality of the evidence was limited. Observational studies have important limitations for causal inference because the association between exposure and behavior could be (partly) due to confounding factors. Studies using respondent recall for both exposure and subsequent behavior are likely to suffer from recall bias. The 1 randomized study was not registered and may not have been successful in blinding the study subjects [34]. The strength of the evidence in the 2 reviews was generally limited as well. Many of the included RCTs did not blind those assessing the outcomes to the treatment in RCTs. The majority of observational studies were cross-sectional making it impossible to infer causality from the association. Finally, the reviews found considerable heterogeneity in study designs and effect measures.

None of the studies included in this review (or in the included reviews) were conducted in Africa and only 2 studies were conducted in Asia. The development of effective policies requires a solid understanding of the exposure to digital marketing and the marketing techniques used across a variety of settings. Studies in more countries are thus needed. Within countries, more evidence is needed on demographic characteristics (that is, socioeconomic status, age, sex, gender) to determine the types of food and drinks that are marketed and the marketing strategies used. None of the studies were based on population-representative surveys.

A key limitation of the available evidence is the accurate assessment of exposure. Many studies relied on self-report which is likely to suffer from reporting and recall bias. In addition, several authors assessed exposure using their own accounts or browsers on incognito mode. This does not provide a valid measure of child and adolescent exposure because content is typically based on personalized algorithms that take into account online behavior such as browsing history, likes, shares, and comments. Algorithms are complex and not publicly accessibly. Better tools, building on real-time screen capture, need to be used to quantify individual exposure to marketing. These tools should not change online behavior while maintaining the study subjects’ privacy. Australian researchers have recently developed SCANNER, an artificial intelligence-enabled system that enables quantifying children’s exposure to and engagement with unhealthy food and drinks in digital environments [63]. Similar efforts are underway in North America [64]. To our knowledge, no studies have used these tools to study digital marketing to children.

A final limitation of the evidence is that no studies have looked at the long-term effects of exposure to digital food marketing. These studies are challenging to conduct considering the multiple factors that can affect food choices and diets and because of the difficulty of assessing exposure to digital marketing over extended periods of time.

The second part of our review focused on policies in LAC countries that have been at the forefront of mandatory regulations limiting exposure of minors to (digital) marketing of unhealthy foods and beverages. The LAC experience may thus provide important insights for the development of effective policies in other countries. Current regulations restrict marketing directed to adolescents ≤12, 14, and 16 y of age, depending on the country. To effectively restrict children’s and adolescents’ exposure to digital marketing, regulations should increase the age to 18 y because both the time spent online and the exposure to marketing are highest in the oldest age groups. Even then, children and adolescents would still be exposed to marketing not directly targeting them. Fully protecting children and adolescents would require banning all forms of (digital) marketing of unhealthy foods and beverages.

Most policies were comprehensive and included websites and social media platforms. The digital marketing techniques covered by the regulations, however, were unclear, which may allow companies to exploit loopholes, potentially undermining the effectiveness of the regulations. Although the institution in charge of monitoring the marketing was explicit in the policies, systems for monitoring compliance were missing.

Most policies used the presence of a FOP warning label to define the scope of the marketing regulation. Foods and beverages that fall under the requirement of carrying a FOP warning label provide a good starting point to identify what should be the subject of digital marketing restrictions [59]. Food labeling schemes based on nutrient profiling systems can help regulators and the food industry to streamline implementation of the regulations by simplifying the identification of foods that exceed specific thresholds for added sugars, saturated fat, and sodium (that is, nutrients to limit) [65]. Policy makers should choose a nutrient profiling system that aligns with population dietary patterns and dietary guidelines [66].

What are the implications of the available evidence for policy? Even though the quality of the reviewed studies varied, the evidence suggests that children and adolescents are subject to significant and growing exposure to digital marketing and that this exposure contributes to unhealthy diets. This conclusion is supported by the large and growing digital marketing efforts of the food and beverage industry [19], which would not happen if there was no sizable return on this investment.

Evidence suggests that self-regulation may not be effective because it is not sufficiently restrictive. An evaluation of the United States Children’s Food and Beverage Advertising Initiative identified several loopholes in the definition of advertising, the nutrition standards used, and the age of children who are under the scope of the regulation [67]. A study of the self-regulatory Canadian Children’s Food and Beverage Advertising Initiative (CAI) found that over the 1-y evaluation period, most food and beverage products (90%) from CAI-participating companies advertised on 10 of the most popular websites for children were categorized as high in fats, sodium, sugar, and calories [68].

Adequately protecting children and adolescents from the harmful effects of the marketing of unhealthy foods and beverages may thus require mandatory policies. The WHO, the UNICEF, and other UN agencies have called on member states to regulate the marketing of unhealthy food and non-alcoholic beverages [23,[69], [70], [71]]. The WHO recently released a guideline on policies to protect children from the harmful impact of food marketing [72]. Policies need to ensure that children’s and adolescents’ rights to adequate nutrition, health, privacy, and information are respected, protected, and fulfilled in the digital environment [[70], [73], [73]]. Marketing regulation policies have been shown to reduce the exposure to and the power of marketing of unhealthy food and non-alcoholic beverages to children [26]. The implementation of policies by countries, however, has been extremely slow and limited in scope, focusing mostly on self-regulation with the participation of the food industry [67,74,75].

The rapidly changing digital ecosystem requires policies that are detailed and comprehensive to avoid that marketing efforts simply migrate from more to less regulated channels, platforms, and (digital) media [7]. The global nature of the internet means that children and adolescents can typically access content that is hosted both inside and outside their country of residence. Effective regulation will thus require collaboration between countries. Enforcing regulations is another important challenge. Real-time monitoring is complicated, given that digital marketing activities take place within a “black box,” usually inaccessible to researchers and regulators [76]. The development of technology that can be used by governments to monitor the extent to which online marketing regulations are followed is an important research need. Promising work using artificial intelligence is underway [63,64]. Monitoring mechanisms need to be developed to provide objective data as well as full transparency not only about what was marketed but also on who was originally targeted and who was ultimately exposed [16,30].

## Author contributions

The authors’ responsibilities were as follows – GF, PV, RS: designed the study; GF: performed the literature review; GF, JLL: analyzed data; GF, JLL: wrote the paper; all authors: assisted in the interpretation of results, were involved in the critical revision of the article, and read and approved the final manuscript.

## Data availability

Data described in the manuscript, code book, and analytic code will be made available upon request pending application and approval.

## Funding

This work was supported by a grant to UNICEF from Novo Nordisk. Funders were not involved in the study design, data collection, analysis, interpretation, or writing of this manuscript. GF and JLL were supported by the CGIAR Research Initiative on Sustainable Healthy Diets through Food Systems Transformation (SHiFT).

## Conflict of interest

The authors report no conflicts of interest.
