# Supplementary material for: Digital Marketing of Unhealthy Foods and Non-alcoholic Beverages to Children and Adolescents: A Narrative Review
Source: Curr Dev Nutr. 2025 Jan 13;9(2):104545. doi: 10.1016/j.cdnut.2025.104545 (PMC11880703; doi:10.1016/j.cdnut.2025.104545)
Supplement: Multimedia component 1 [file mmc1.docx]

**Supplementary Table 1. Keywords**

| **Keywords** | Connectors: AND, OR. |
| --- | --- |
| Keywords (English) | Related to marketing: marketing, digital, advertising, direct-to-consumer advertising, advert*, advergame*, sponsor*, promot*, market*, adspend*, commercial, commercials  Population: child*, adolescent*, infant*, youth*, young people, young person, teen*, pupil*  Nutrition: diet*, snack*, nutrition, fast food, beverage*, carbonated beverages, energy drinks, milk, milk substitutes, drink*, food*, food industry  Settings: preschool, school |
| Keywords (Spanish) | Related to marketing: *marketing, marketing* digital, publicidad, publicidad dirigida al consumidor, public*, advergame*, aupiciante, promoc*, mercad*, adspend*, comercial, comerciales  Population: niñ*, adolescent*, infant*, jóven*, personas jóvenes  Nutrition: alimentación, snack*, nutrición, comida rápida, bebida*, bebidas carbonatadas, bebidas energizantes, leche, substitutos de la lecha, bebida*, alimento*, industria alimentaria  Settings: pre-escolar, escuela |

**Supplementary Table 2. Inclusion criteria**

| **Criterion** | **Inclusion** |
| --- | --- |
| Year of publication | Publications between January 2012 and July 2024 |
| Type of publication | Scientific articles that underwent peer review |
| Language | Spanish or English |
| Population | Children and adolescents from 2 to 18 years of age |
| Outcome variables | Exposure, food preference, food choice, food intake |
| Theme | Publications that: (1) describe the exposure or power of digital marketing of unhealthy foods and beverages to children and adolescents, (2) identify elements to consider in the design of unhealthy food and beverage digital marketing restriction policies, (3) evaluate the effect of unhealthy food and beverage digital marketing on children and adolescents’ outcomes |
